# CRISPR/Cas9: A Robust Genome-Editing Tool with Versatile Functions and Endless Application

**DOI:** 10.3390/ijms21145111

**Published:** 2020-07-20

**Authors:** Baohong Zhang

**Affiliations:** Department of Biology, East Carolina University, Greenville, NC 27858, USA; zhangb@ecu.edu

Since a potential genome editing tool was first recognized in 2012 [1,2], the CRISPR/Cas9 system has been becoming a powerful and robust genome editing tool for gene function study and crop improvement. Over the past decade, as new Cas enzymes have been identified, current Cas9 enzymes modified, and new bioinformatics tools developed, CRISPR/Cas9-based research has been developing extremely quickly. Particularly, the modification of Cas enzymes has significantly boosted the application potentials of CRISPR/Cas9 genome editing [3]. Although there are many other Cas enzymes identified and utilized in genome editing, the CRISPR/Cas9 system currently refers to any CRISPR/Cas system, including Cas12 and Cas13, majorly due to the fact that Cas9 is the first and most commonly used Cas enzyme in genome editing [4]. At present, CRISPR/Cas9 genome editing has been widely used in many plant species, including both model plant species and agriculturally important crops, such as wheat [5], cotton [6] and soybean [7], not only for gene functional study but also for crop improvement. To facilitate the quick development and application of CRISPR/Cas9 genome editing. We edited this Special Issue of “Genome Editing in Plants”. In a short time period, this Special Issue attracted a lot of attention from the scientific field and industries. Finally, after the expert peer review, a total of 15 papers were accepted to be published in this Special Issue of *International Journal of Molecular Sciences*.

Among the 15 published papers, three are timely review papers. Jansing and colleagues (2019) reviewed the technical and practical consideration of genome editing in agriculture, particularly focusing on the current delivery method of the CRISPR/Cas9 system into plant cells and their regeneration methods. They also discussed the suitability of CRISPR/Cas9 for improving different traits of agriculturally important crops [8]. Among all crops, significant progress has been made in rice using CRISPR/Cas genome editing technology. In a review written by Fiaz and colleagues (2019), they reviewed the current status of CRISPR/Cas9 on rice improvement, particularly on rice grain quality improvement [9]. Increasing CRISPR/Cas9 on-target efficiency and reducing the off-target effect is always a major topic for genome editing. A lot of offers have been added into this field in the past half-decade. In this Special Issue, Hajiahmadi and colleagues (2019) reviewed the major strategies for reducing the off-target effect of CRISPR/Cas9. They thought that the combination of single guide RNA (sgRNA) and a ligand-dependent aptazyme strategy may be a great strategy for decreasing the frequency of off-target mutations in plants [10].

The remaining 12 research articles are associated with 12 different plant species, including rice [11], cotton [12], wheat [13,14], *Brassica napus* [15], soybean [16], sweet potato [17], cowpea [18], tomato [19], potato [19], chicory [20] and model plant *Arabidopsis* [21], as well as alga *Chlamydomonas reinhardtii* [22]. From here, it is clearly seen that more and more research has been focused on agriculturally important crops. The majority of these studies employed traditional CRISPR/Cas9 technology to knock out an individual gene, except one study, which employed the CRISPR/Cas9 base editor to create a transgene-free genome editing tomato and potato [19]. Among these studies, the majority of them worked on agriculturally important traits. For example, Wang and colleagues (2019) studied the function of *IbGBSSI* and *IbSBEII* genes in starch biosynthesis in sweet potato by CRISPR/Cas9 genome editing [17]. Chen and colleagues investigated the function of the M-locus protein kinase (*MLPK*) *BnaMLPKs*, the functional homolog of *BrMLPKs* in *Brassica rapa* [15]. Chen and colleagues used *Arabidopsis* as a model to study the role of *DPA4* and *SOD7* genes in seed development and response to abiotic stress [21]. All these studies have provided new tools and/or new insight into our understanding of agriculturally important traits.
